# Light repair effect of polysaccharides component from white *Ganoderma lucidum* and *Laminaria japonica* fermentation broth attenuates epidermal barrier dysfunction via TRPV4-Keap-1/Nrf2 pathway

**DOI:** 10.1186/s40643-025-00949-7

**Published:** 2025-10-10

**Authors:** Xinrui Jiang, Qianru Sun, Yunxia Chen, Ning Su, Jiaxuan Fang, Zixin Song, Meng Li, Changtao Wang, Dongdong Wang

**Affiliations:** 1 College of Light Industry Science and Engineering, Beijing Technology and Business University, 11 Fucheng Road, Haidian District, Beijing, 100048 People’s Republic of China; 2 Chinese Academy of Inspection and Quarantine, Beijing, People’s Republic of China

**Keywords:** *Laminaria japonica*, Fermented polysaccharides, Epidermal photoinflammation, Skin barrier, TRPV4-Keap-1/Nrf2 signaling pathway, Ganoderma lucidum

## Abstract

Epidermal photoinflammation has emerged as an atypical pathological response that poses a growing global threat to human health and well-being. In this study, a polysaccharide component (named LJGp) was isolated from *Laminaria japonica* and white *Ganoderma lucidum* fermentation broth, and its reparative effects on UVB-induced epidermal damage were investigated. LJGp was found to restore the migration ability of HaCaT cells, increase antioxidant enzyme activities, and clear excessive intracellular ROS. At 1500 μg/mL, ROS levels were reduced to about 50% of those in the UVB group, while IL-6 expression decreased to a level comparable with the control group. At the same time, LJGp increased barrier functional proteins, inhibited desmosome degradation, and maintained the stability of the epidermal barrier. Mechanistic results further showed that LJGp regulated the TRPV4-Keap-1/Nrf2 pathway, where UVB-induced TRPV4 activation suppressed Nrf2 activity and promoted AP-1–mediated inflammation. By reducing TRPV4 overexpression and restoring Nrf2 function, LJGp alleviated barrier dysfunction and improved epidermal repair. These results provide supporting data and a scientific basis for the development and utilization of LJGp as an epidermal photoinflammatory relief agent.

**Graphical abstract:**

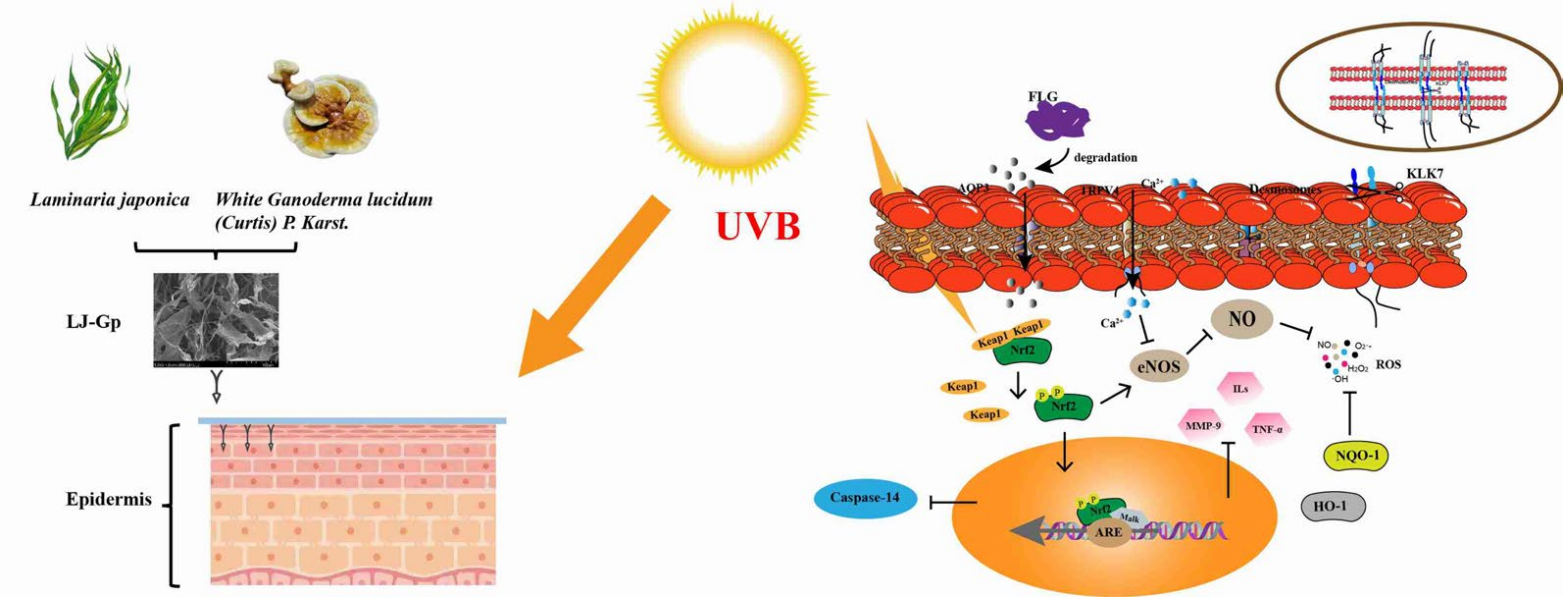

**Supplementary Information:**

The online version contains supplementary material available at 10.1186/s40643-025-00949-7.

## Introduction

Skin, as the organ with the largest surface area, serves as the first defense barrier against environmental insults and microbial invasion (Grice and Segre 2011). Its protective role primarily relies on the stratum corneum (SC), which consists of terminally differentiated keratinocytes rich in keratin proteins (Eckhart et al. 2013). Continuous keratinocyte renewal maintains epidermal homeostasis (Cibrian et al. 2020), but excessive external stimuli can accelerate damage to the integrity of the original barrier structure of the epidermal layer, disrupt the balance of replacement, and lead to the development of inflammatory skin diseases (Darido et al. 2016). Solar radiation is one of the direct factors that can cause harmful effects on the skin (Krutmann et al. 2017). UVB (320–280 nm) mainly stays in the epidermal layer and can burn the skin in a short period of time, for which it is considered to have a stronger damaging effect than UVA (Ryu et al. 2015). In 2017, the World Health Organization classified UVB as a Class I carcinogen. Excessive exposure to UVB can lead to the occurrence of epidermal photoinflammation, with macroscopic manifestations including sunburn, pain, itching, and edema (Sun et al. 2022). While current studies mostly emphasize skin repair after sunburn, the changes in a series of physiological signals caused by pain after sunburn are ignored.

Previous studies have confirmed that a large number of chemokines produced by epidermal inflammatory response can also cause and promote pain (Wei et al. 2012). Transient receptor potential (TRP) ion channels, especially TRPV4, can be activated by multiple pathways and are involved in inflammation-induced pathological pain sensitization (Chen et al. 2013; Ehlers et al. 2023). UVB can enhance the expression of TRPV4 in keratinocytes, confirming that it is involved in the UVB-induced sunburn pain response. In addition, TRPV4 has also been confirmed to participate in the inflammatory response. This reaction process is accompanied by immune cell recruitment and changes in the epidermal structure, and a large amount of interleukin-6 (IL-6) is produced. The above results revealed that the TRPV4 channel can be selectively activated by UVB and become the initiating target of epidermal photoinflammation. It relies on extracellular calcium influx to cause itch, pain, and an inflammatory infiltration reaction (Moore et al. 2013). Nrf2 is a transcription factor with nuclear translocation activity that controls genes related to cellular detoxification and oxidative stress protection. It is involved in the regulation of inflammatory response and epidermal barrier function differentiation during wound healing (Frantz et al. 2023). In addition, mice with impaired Nrf2 expression were found to be more susceptible to actinic keratosis, confirming that the positive response of the Nrf2 protein provides a solid defense against UV radiation for the epidermis (Saw et al. 2014; Schäfer et al. 2010). The key target genes and proteins regulate multiple biological signals. These findings indicate that TRPV4 activation and Nrf2 signaling are central to the initiation and regulation of UVB-induced photoinflammation. However, the interplay between pain signaling and epidermal inflammation remains insufficiently understood.

As a result of the spread and deepening of the concept of sustainability, the exploitation of marine resources has increased. The sulfated polysaccharides, polypeptides, and polyphenols contained in algae have many biological activities. *Laminaria japonica*, also known as kombu, is a global economic algae. About 4.8 million tons of dried *L. japonica* can be harvested in China's coastal areas every year, but most of it is used to make animal feed or discarded after simple primary processing, resulting in a great waste of resources (Luan et al. 2021). *L. japonica* is rich in fucoidan, fucoxanthin, and polyphenols. Fucoidan is the most abundant component in *L. japonica*, representing a class of biomacromolecules with special physiological activities (Jia et al. 2014). However, the complete extraction of active substances is hampered by the inherent hard cell wall, which cannot be fully extracted and separated (Cao et al. 2016; Wang et al. 2008). Fermentation is a widely used food processing technology that can improve the bioavailability of nutrients. Fermentation can change the proportion of nutrients in food and the physical properties of some active substances (Kim et al. 2013). Microbial fermentation raw materials can be used to obtain value-added products, which can promote the hydrolysis of polymers in raw materials, the synthesis of bioactive molecules, or the degradation of toxic factors (Sadh et al. 2018).

Previous results (Sun et al. 2022) showed that the three kinds of *L. japonica* fermentation broth could alleviate the radiation damage effects of UVB on human epidermal cells. Considering the requirements of bioavailability and physiological activity, the most abundant fermented polysaccharides were isolated and their physical properties identified. The results showed that fermented polysaccharides had a special micro morphology, molecular weight, and monosaccharide composition.

However, it is still unclear whether there is a link between the difference in polysaccharide composition and the efficacy of fermentation broth. This study aimed to evaluate the correlation between the efficacy of fermentation broth and polysaccharide composition, established a UVB damage repair model of human immortalized keratinocyte (HaCaT) cells to explore the biological activity of polysaccharides, and significant signaling changes in the processes regulating the onset of photoinflammation, aiming to provide scientific data and theoretical support for the application of fermented polysaccharides as an epidermal photoinflammatory relief agent. In particular, we hypothesize that the combination of *G. lucidum* and *L. japonica* fermented polysaccharides provides a novel and synergistic strategy to attenuate UVB-induced photoinflammation through modulation of TRPV4 and Nrf2 pathways, thereby distinguishing this study from prior reports on single-source polysaccharides.

## Materials and methods

### Materials source and chemicals

*L. japonica* was produced in Rongcheng, Dalian, China. It was dried, crushed, and screened (50 mesh) for future use. Potato dextrose agar (PDA) media were purchased from a Beijing chemical plant (Beijing, China). *G. lucidum* came from laboratory preservation (number: CGMCC No.17789). HaCaT cells were purchased from Beijing United Medical College (Beijing, China). 0.05% (containing EDTA) trypsin and double antibody were purchased from GIBCO (California, USA). CCK-8, First-Strand cDNA Synthesis, and Fast Super EvaGreen® qPCR Master Mix kits were purchased from Biorigin Biotechnology Co., Ltd. (Beijing, China). A HO-1 kit was purchased from Jiancheng Bioengineering Institute (Nanjing, China). ILs, TNF-α, and FLG kits were purchased from Wuhan Huamei Bioengineering Co., Ltd. (Wuhan, China). INV and LOR ELISA kits were purchased from Cloud-Clone Technology Co., Ltd. (Wuhan, China). ROS detection kit, DAPI nuclear dye solution, and PMSF were purchased from Beyotime Biotechnology Co., Ltd. (Shanghai, China). TriQuick total RNA extraction reagent was purchased from Solarbio Biotechnology Co., Ltd. (Beijing, China).

### Sample preparation

The sample preparation and the operation procedures for extracting and separating polysaccharides follow the previous research methods (Sun et al. 2022) with minor modifications. White *G. lucidum* colonies were picked out and cultured in PDA solid media at 28 °C for 10 days, then inoculated into PDA liquid media, and cultured at 28 °C for 7 days to obtain mycelial pellets. *L. japonica* was dissolved in deionized water at a ratio of 1:40 and sterilized at 121 °C for 30 min. 5% of *G. lucidum* was added, and the solution was shaken at 28 °C and 180 r/min for 7 days. The supernatant was obtained by centrifugation at 4800 r/min for 30 min to obtain the fermentation broth of *G. lucidum*, which was named “LJG”.

The polysaccharides in LJG were separated using alcohol precipitation (3 volumes of 95% ethanol, overnight at 4 °C). The precipitate was replenished to the original volume with water, and the protein was eluted with Savage reagent. Collected precipitate, dissolved in ultrapure water, and dialyzed (up to 10 kDa MW) for 48 h to obtain water-extracted polysaccharides and fermented polysaccharides, which were named “LJGp”, freeze-dried for future use.

### Cytotoxicity assay

Cells cultured under the same conditions without UVB irradiation and without LJGp treatment were designated as the blank control group (C group). According to the reference (Fang et al. 2023), cell viability was determined by CCK-8 colorimetry. The HaCaT cells were digested with trypsin until they fell off. They were then seeded into 96-well plates at a density of 1 × 10^4^ cells/well and cultured for 24 h at 37 °C in a humidified incubator of 5% CO_2_. 100 µL of LJGp samples dissolved in DMEM basic medium with different concentrations were added to each column of wells in the sample group (the control group was replaced by DMEM basic medium), and the cells were cultured for 12 h. The supernatant was aspirated, an equal amount of DMEM medium was added to each well, 10 µL of CCK8 reagent was added, the samples were incubated for 2 h, and the OD_450_ was determined. The survival rate of the HaCaT cells was calculated according to the following formula: cell survival rate (%) = sample group OD_450_/control group OD_450_ × 100%.

According to the damage dose of UVB in our previous research (Sun et al. 2022), the cells cultured in 96-well plates for 24 h were irradiated with different doses (0, 5, 10, 20, 30, 40 mJ/cm^2^) of UVB under the ultraviolet spectrometer. The total UVB energy was set at 40 mJ/cm^2^, and the 96-well plate was covered with a double layer of tin foil. PBS (1 ×, pH 7.4) was added between the cells in each column to prevent intergroup interference. The position of the tin foil was adjusted during irradiation until the energy was exhausted. The remaining steps were the same as above.

### Determination of cell migration ability

Determination of cell migration ability is based on the references (Cha et al. 2014). The HaCaT cells (30 × 10^4^/well) were cultured in 6-well plates. Scratched the parallel line of each hole 3 times and irradiated UVB. Each well was added with different concentrations of LJGp (the model group was replaced with the same amount of DMEM basal medium). The healing degree of the cell scratch was observed at 0 h and 24 h, and the percentage of healing area between cell scratches was calculated.

### Determination of ROS and JC-1

The HaCaT cells (30 × 10^4^/well) were cultured in 6-well plates. The ROS content of the HaCaT cells damaged by UVB was determined by the ROS kit and reference (Su et al. 2022), and the fluorescence intensity was recorded. The control group and model group (equal amount of DMEM basic medium) were operated at the same time as the sample group to ensure consistent treatment conditions.

The mitochondrial membrane potential of HaCaT cells was measured by using the Enhanced Mitochondrial Membrane Potential Detection Kit (Beyotime, Shanghai, China, C2003).

### Enzyme-linked immunosorbent assay (ELISA)

ELISA techniques have been widely applied for cytokine and protein detection, as reported previously (Abd El-Aziz and Younes 2019). In the present study, a commercial sandwich ELISA kit was employed to ensure high sensitivity and specificity.

The HaCaT cells (30 × 10^4^/well, 2 mL/well of culture solution) were cultured until the degree of adherence reached 80%. 2 mL of PBS buffer was added to each well. After the damage, the supernatant was taken for standby.

The IP lysate was mixed with PMSF at a ratio of 100:1. The culture solution in the 6-well plate was sucked up and washed twice with PBS buffer. 100 µL of cell IP lysate was added to each well, and the well plate was shaken to ensure that the lysate made full contact with the cells at the bottom. The cells at the bottom of the plate were scraped off after about 30 s, the cell lysate and precipitation mixture were collected and centrifuged at 10,000 r/min for 10 min, and the supernatant was obtained. A BCA protein concentration assay kit (Beyotime, Shanghai, China) was used for protein calibration, following the manufacturer’s instructions.

### IF staining

The IF staining was carried out according to the method described in (He et al. 2019). The HaCaT cells were fixed with a 4% paraformaldehyde solution. Triton X-100 reagent was used to increase the permeability of the cell membranes (Mattei et al. 2017). BSA was used for the sealing operation. The primary antibody was incubated overnight at 4 °C. The diluted fluorescent secondary antibody was added in the dark at a dilution ratio of 1:500 and incubated for 1.5 h. Light was avoided, and a DAPI dye solution was added. An anti-fluorescence quenching agent was then added, the bottom plate was covered, and the protein fluorescence staining was observed under a fluorescence inverted microscope.

### Quantitative reverse transcription PCR (RT-qPCR)

Total RNA was extracted using Trizol and First Stand cDNA for reverse transcription. Refer to the instructions for the specific operation steps. See Table S1 for the reverse transcription reaction system. PrimerExpress was used to design the gene primer sequences, and the β-actin gene was used as the internal reference gene. See Table S2 for the detailed primer sequences. A Fast Super EvaGreen® qPCR Master Mix was used for detection. The qRT-PCR reaction system is shown in Table S3. The reaction cycle parameters were set according to the instructions.

### Statistical analysis

Statistical analysis was carried out using Prism 8 (GraphPad, San Diego, CA). The images were processed using Prism 8 (GraphPad, San Diego, CA), ImageJ v1.8.0 (National Institutes of Health, Bethesda, MD), and Illustrator 2021 (Adobe, San Jose, CA). We have specified the use of untreated control and UVB-only model groups for all assays. Each experimental group included at least three biological replicates, and data were analyzed using one-way ANOVA and expressed as mean ± standard deviation. The correlation analysis was mapped using ChiPlot (https://www.chiplot.online/gene_cluster.html). Statistical significance was determined at a significance level of *P* < 0.05.

## Results

### Correlation analysis of polysaccharide composition and biological activity in fermentation broth

**Fig. 1:**
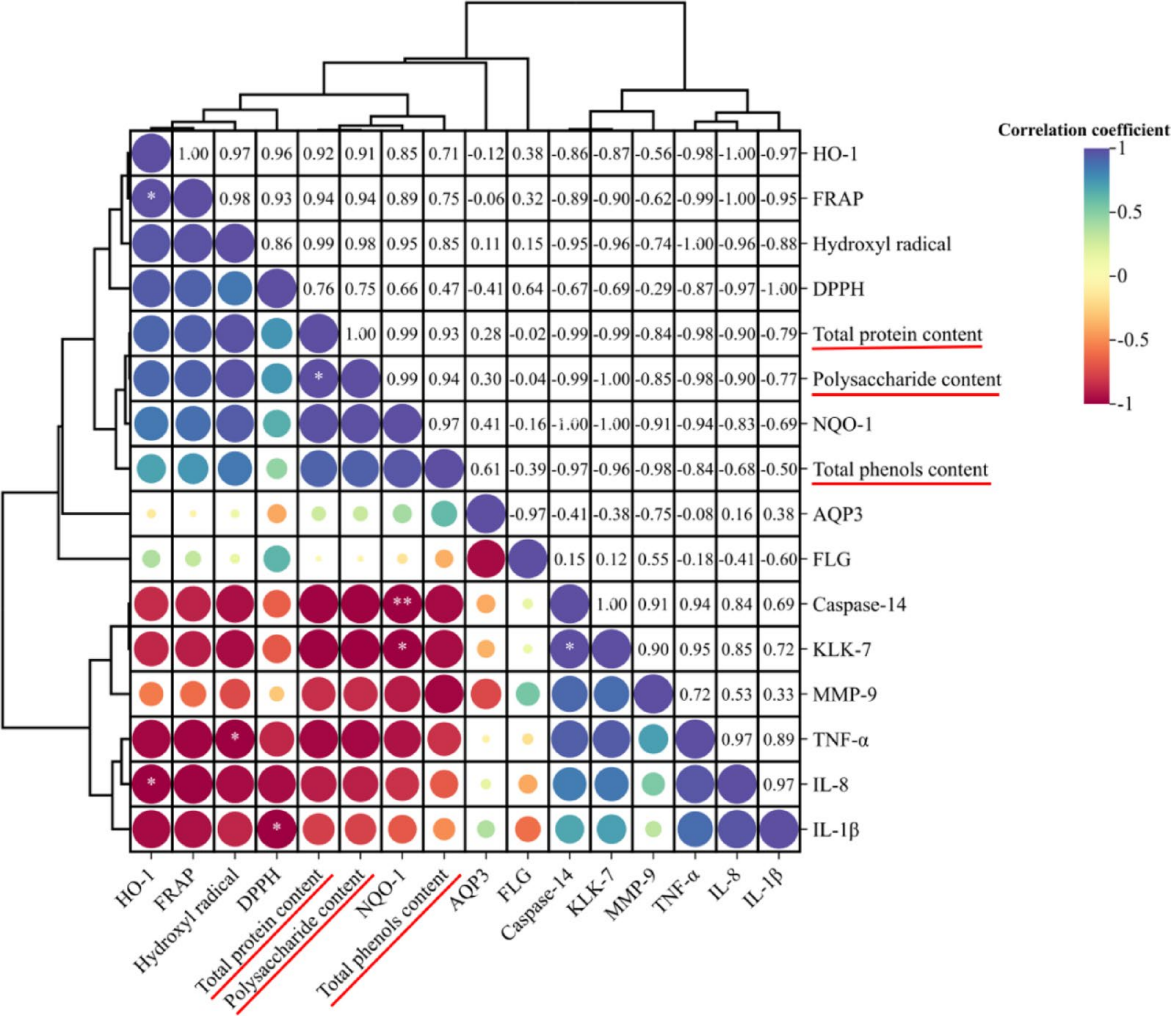
Correlation analysis of active substances and bioactive functions in *Laminaria japonica* fermentation broth. Statistical annotations: **P < 0.01, *P < 0.05

Correlation analysis was used to evaluate the correlation between the content of different active substances in *Laminaria japonica* fermentation broth and efficacy (Fig. 1). The size and color of the dots in the figure indicate the difference in correlation between the two indicators. The correlation between total protein, polysaccharide, and total phenol and efficacy indexes was statistically compared. The data showed that the correlation between total phenols and photoinflammatory repair activity was weak. The correlation between total proteins and polysaccharides was similar. Strong correlation indices were distributed in antioxidant, inflammatory, and barrier functional enzymes. The hydroxyl radical scavenging ability and NQO-1 content were strongly positively correlated with polysaccharides and proteins. While TNF-α, KLK-7, and Caspase-14 were negatively correlated.

Previous data (Sun et al. 2022) showed that the content of polysaccharides in kelp fermentation broth was significantly higher than that of total protein. The comprehensive remediation effect of white *Ganoderma lucidum Laminaria japonica* fermentation broth is better than that of *Laminaria japonica* yeast fermentation broth. Combined with the results of correlation analysis, we chose to extract polysaccharide components from kelp fermentation broth for further study.

### LJGp promotes cell migration and inhibits apoptosis

The slow proliferation and differentiation, and abnormal apoptosis caused by damaged epidermal cells are the direct factors for the weakening of barrier function. The study evaluated the negative effects of HaCaT caused by UVB radiation and whether LJGp could alleviate such effects.

**Fig. 2:**
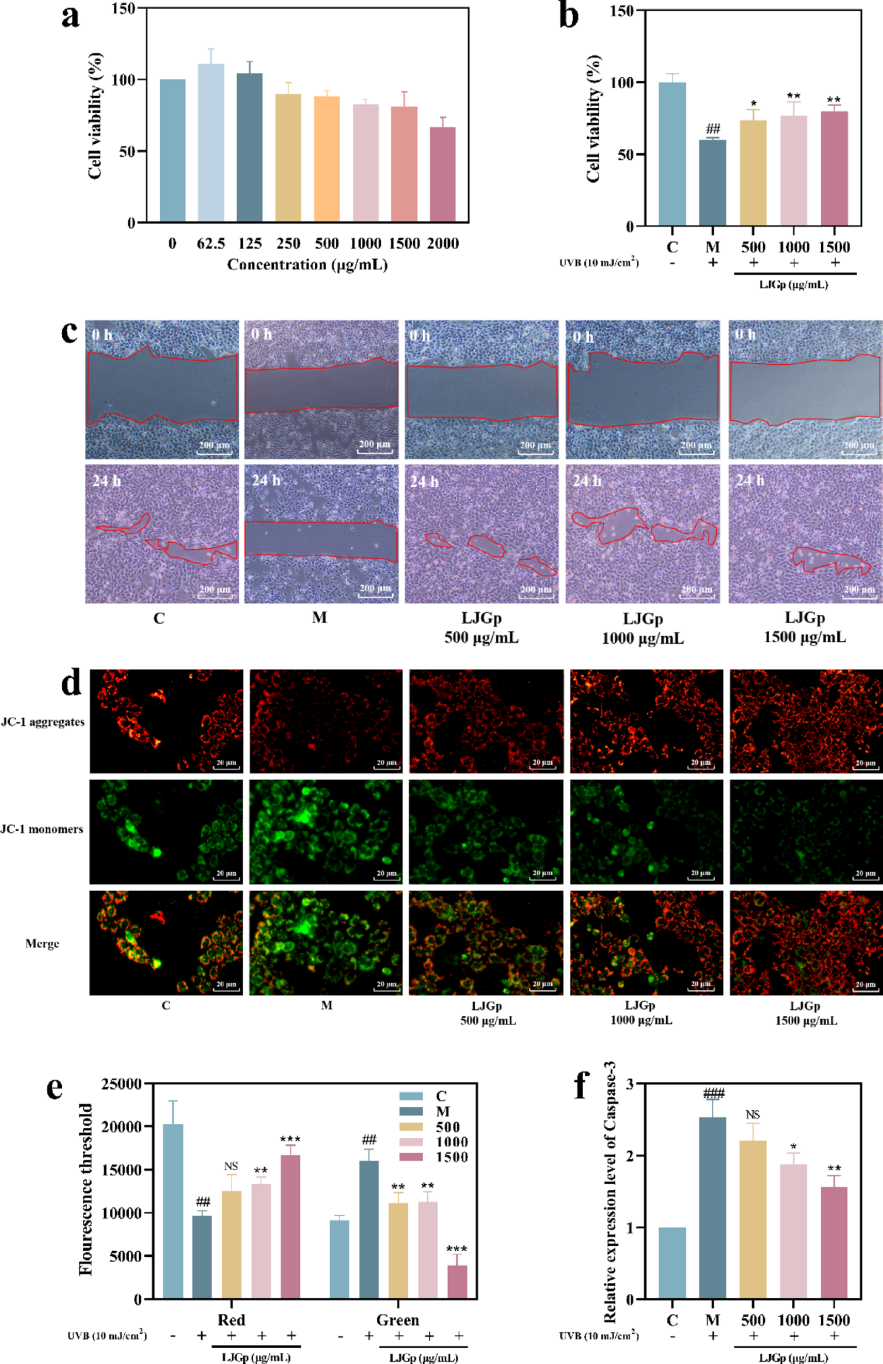
Proliferative toxicity and migration-promoting function of LJGp on HaCaT cells. **a** Proliferation toxicity on normal HaCaT cells. **b** Proliferation toxicity of HaCaT cells irradiated by UVB. **c** Differences in the scratch wound healing area. Scale bars = 200 μm. **d** Fluorescence images of JC-1 monomers (green) and aggregates (red). Scale bars = 20 μm. **e** Fluorescence values of JC-1 monomers and aggregates. **f** The content of pro-apoptotic protein Caspase-3. Statistical annotations: ***P < 0.001, **P < 0.01, *P < 0.05, *ns* (not significant, *P* > 0.05). Symbols ###, ## indicate comparisons with the model group

With the increase of concentration, the viability of HaCaT cells decreased gradually. When the mass concentration was 1500 and 2000 μg/mL, the cell viability decreased to 81% and 67% respectively. It was determined that 1500 μg/mL was the non-toxic maximum concentration of LJGp on uninjured HaCaT cells. LJGp promoted HaCaT proliferation, and the survival rate reached 111% (Fig. 2a).

Therefore, concentrations of 500, 1000, and 1500 μg/mL were selected for subsequent exploration.

The effects of LJGp on the survival rate of damaged HaCaT cells are shown in Fig. 2b. The data shows that the cell survival rate was positively correlated with the concentration. Compared with the model group, the cell survival rates were significantly improved. It reached its maximum at 1500 μg/mL LJGp, and the cell survival rate was 80%.

The scratch test was used to explore the effects of LJGp on the migration function of injured cells (Fig. 2c**, **Table 1). After 24 h of cell growth, the scratch blank area in the control group accounted for 16%, while that in the model group accounted for 76%. The observation pictures and calculation results show that HaCaT after light damage lose their original migration and differentiation abilities, and cannot supplement new cells to the blank site in time. Compared with the model group, the proportion of the scratch blank area decreased significantly after treatment with LJGp for 24 h, which was 13% (500 μg/mL), 21% (1000 μg/mL), and 10% (1500 μg/mL). When the sample concentration was 1500 μg/mL, the proportion of the blank area reached its minimum and was smaller than that of the control group. The above data confirms that LJGp effectively repaired the weakening of epidermal cell migration caused by UVB radiation. What’s More, 1500 μg/mL LJGp can significantly restore the migration ability of cells on the basis of repairing the damage, and even exceed the unimpaired level.

**Table 1:** Changes in scratch area of HaCaT after treatment with different concentrations of LJGP

	Control	Model	500	1000	1500
0 h	9.3 ± 1.6	17.7 ± 3.1	8.4 ± 1.3	10.8 ± 2.4	9.8 ± 1.3
24 h	1.5 ± 0.5	13.4 ± 2.1	1.1 ± 0.4	2.2 ± 0.2	1.0 ± 0.3
Area (%)	16	76	13	21	10

In addition to affecting the survival state of cells, UVB irradiation can also lead to apoptosis (Ding et al. 2023). The mitochondrial membrane potential of intact cells is relatively stable, a decrease in mitochondrial transmembrane potential is an early event in the cascade of apoptosis (Iwata et al. 2002). Due to its ability to specifically cleave poly ADP ribose polymerase (PARP1) and acetyl-DEVD-7-amino-4-methylcoumarin (Ac-DEVD-AMC), Caspase-3 can cleave cell DNA and then cause cell apoptosis (Zhang et al. 2023). Therefore, changes in mitochondrial membrane potential and a special increase in Caspase-3 content are indicators used to evaluate early apoptosis.

The real-time membrane potential of mitochondria was detected using a JC-1 fluorescent probe. When the membrane potential is high, JC-1 gathers in the mitochondrial matrix and emits red fluorescence. When the membrane potential is low, JC-1 cannot gather and mostly exists in the form of a monomer, emitting green fluorescence. The JC-1 fluorescence measurement results (Fig. 2d, e) show that the red fluorescence intensity of mitochondria in the control group was high, while the green fluorescence intensity was low, indicating that the mitochondrial membrane potential was high at this time. Compared with the control group, the green fluorescence intensity in the model group was significantly increased, the content of the JC-1 monomer was higher, and the mitochondrial membrane potential was significantly decreased. After LJGp treatment, the red fluorescence intensity in the mitochondria of damaged cells increased significantly, indicating that the content of the JC-1 monomer was high and the mitochondrial membrane potential had returned to a higher level.

The Caspase-3 detection results showed that the protein content of the model group was significantly higher than that of the control group, indicating that UVB irradiation caused cells to secrete more Caspase-3. In addition to 500 μg/mL, the relative content of Caspase-3 in the sample group decreased significantly, and it in the sample group decreased significantly at 1500 μg/mL, this confirmed that LJGp effectively reduce the abnormal cell apoptosis caused by UVB irradiation (Fig. 2f). More importantly, after the action of high concentration LJGp, the relative content of Caspase-3 in cells remained higher than the undamaged level, which is consistent with JC-1.

The above experimental results show that a certain concentration of LJGp can improve the proliferation activity and migration function of HaCaT cells damaged by UVB without affecting their normal growth. UVB irradiation can cause a decrease in mitochondrial potential and excessive synthesis of Caspase-3, promoting DNA cleavage and leading to the apoptosis process of epidermal cells. LJGp effectively alleviated the degree of epidermal cell apoptosis and repaired the structural damage of epidermal cells caused by ultraviolet radiation.

### LJGp inhibits oxidative stress

Oxidation and inflammatory factor accumulation can cause keratinocyte dysfunction, infiltrating cells, and change the original health state of the ECM.

Under normal circumstances, ROS participates in biochemical reactions in cells to maintain body functions. Stimulation by ultraviolet radiation can lead to the production of a large amount of ROS in a short time. Excessive ROS will cross the cell membrane and combine with a variety of biological macromolecular substances, including DNA, to produce an oxidative reaction, which will cause oxidative stress and an inflammatory reaction. Heme oxygenase-1 (HO-1) is an antioxidant, cell protective enzyme that can catalyze the oxidative degradation of heme (Piantadosi et al. 2008). NAD(P)H quinone oxidoreductase 1 (NQO-1) is a cell Phase II detoxification enzyme that can protect the cell membrane from ROS attack (Dinkova-Kostova et al. 2005). ENOS is a Ca^2+^ inhibitory NO synthase that can promote the production of NO in cells and play a cardiovascular protective role, and also has ROS synthesis inhibitory activity. Studies have found that the secretion of NO can inhibit the production of ROS and alleviate the oxidative stress response of cells (Hao et al. 2022).

**Fig. 3:**
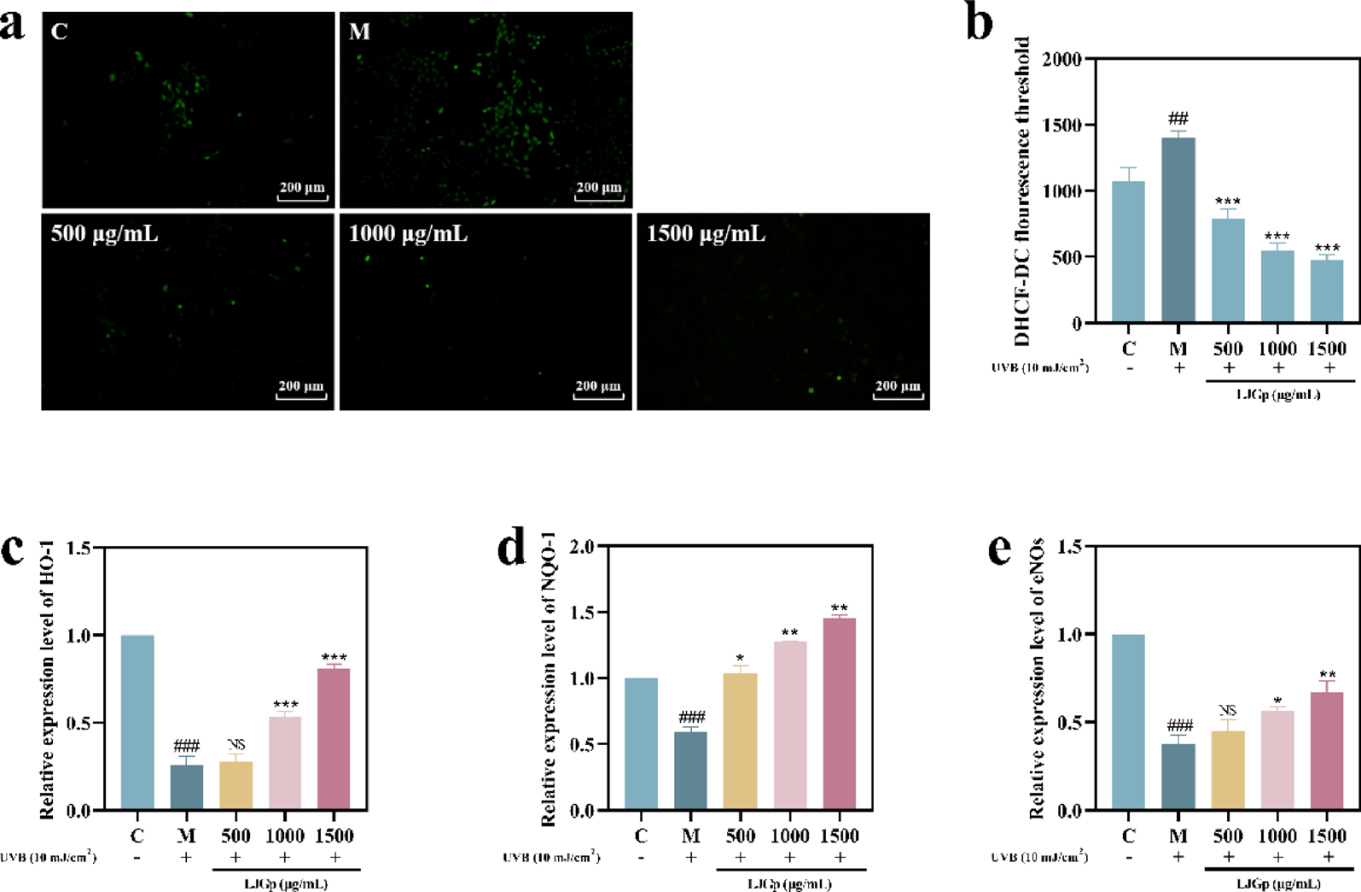
Scavenging effect of LJGp on intracellular ROS and promotion of antioxidant enzyme activities. **a** Intracellular ROS fluorescence observation diagram and intensity. Scale bars = 200 μm. **b** Intracellular ROS fluorescence intensity. **c** HO-1 enzyme activity. **d** NQO-1 enzyme activity. **e** eNOs enzyme activity. Statistical annotations: ***P < 0.001, **P < 0.01, *P < 0.05, *ns* (not significant, *P* > 0.05). Symbols ###, ## indicate comparisons with the model group

Figure 3a and b show that the fluorescence brightness and intensity of intracellular ROS in the model group were significantly higher than those in the control group. After treatment of the sample, the fluorescence intensity decreased significantly and reached its lowest at 1500 μg/mL, about half of the undamaged level.

Compared with the control group, the activities of NO-1, NQO-1, and eNOS in the model group were significantly decreased (Fig. 3c-e). After LJGp treatment, the activity of antioxidant enzymes and the level of transcriptional synthesis were significantly increased in a dose-dependent manner. The activity of three antioxidant enzymes in the sample group reached the highest point at 1500 μg/mL, approximately 1–3 fold that of the model group, and the transcription level was about 2–4 fold higher. At concentrations of 1000 and 1500 μg/mL, NQO-1 enzyme activity was higher than that of the control group. The above data show that UVB irradiation causes the excessive accumulation of ROS in epidermal cells and consumes a large number of antioxidant enzymes. LJGp effectively promoted the synthesis and transcription level of protein, rapidly supplemented the consumed antioxidant enzymes, and alleviated the imbalance of intracellular ROS dynamic clearance.

The above experimental results confirm that UVB irradiation causes oxidative stress injury in epidermal cells. LJGp can remove excessive intracellular ROS, alleviate the oxidative damage of the epidermis.

### LJGp inhibits epidermal inflammation

**Fig. 4:**
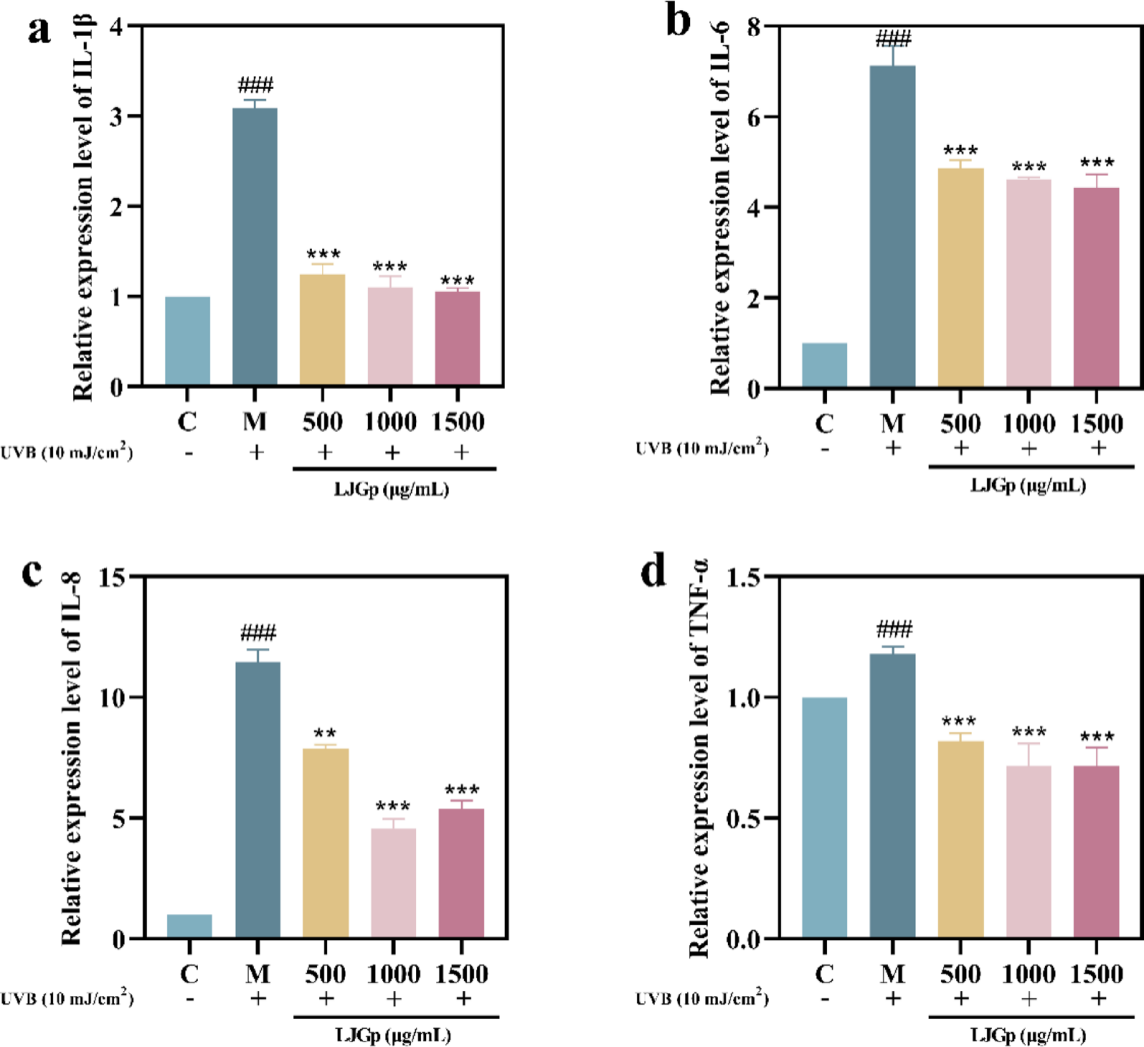
Inhibitory effects of LJGp on intracellular inflammatory chemokines. **a** IL-1β content. **b** IL-6 content. **c** IL-8 content. **d** TNF-α content. Statistical annotations: ***P < 0.001, **P < 0.01. Symbols ### indicate comparisons with the model group

UVB radiation can also cause the large secretion of inflammatory chemokines in cells, leading to the infiltrative inflammatory response of epidermal cells. LJGp has an inhibitory effect on the content and transcription level of cytokines (Fig. 4a-d). Compared with the control group, the content and transcription levels of ILs and TNF-α were significantly increased. The levels in the sample group were significantly lower than those of the model group. Except for IL-8, the contents of other pro-inflammatory factors were dose-dependent and reached their lowest level at a concentration of 1500 μg/mL. After 1500 μg/mL LJGp treatment, the mRNA transcription levels of pro-inflammatory factors were significantly decreased, and the decrease of IL-6 was close to the uninjured level.

The above experimental results confirm that fermented polysaccharides can inhibit the synthesis and release of pro-inflammatory factors and alleviate the inflammatory reaction.

### LJGp regulates epidermal barrier differentiation proteins and active enzymes

Filaggrin can form a close physical barrier in the epidermal tissue to prevent the loss of water and the invasion of external stimuli. Water-retaining proteins in cells play an important role in maintaining the epidermal barrier function (Sandilands et al. 2009). Aquaporin-3 (AQP-3) is a mosaic protein of the cell membrane that is responsible for binding skin keratin and controlling the transfer of water molecules (Zhang et al. 2022). Along with FLG and AQP3, involucrin (INV) and loricin (LOR) are the main functional proteins that constitute the epidermal defense barrier. INV can form a unique cross-connection structure (cuticular membrane) with LOR and act as a barrier defense structure in the epidermal spinous layer (Zhang et al. 2023). Kallikrein can cut off the desmosomes of intercellular junctions, which can lead to abnormal shedding of cells and damage the epidermal barrier function (Zhang et al. 2022). When the barrier function is impaired, the epidermal defense and water retention functions decrease. The loss of the intercellular natural moisturizing factor (NMF) is accelerated, and caspase-14 and FLG need to be consumed to supplement the NMF. UVB radiation can also cause the excessive secretion of matrix metalloproteinase-9 (MMP-9), which is one of the mediators of the vicious cycle of inflammation, responsible for the degradation of various components of the extracellular matrix, including the epidermal functional proteins (Sun et al. 2022). The excessive consumption of FLG and other components will disrupt the tight physical barrier of the epidermis, leading to a vicious cycle of barrier dysfunction. The organic coordination between these structural and functional proteins and enzymes is very important to maintain the function of the epidermal barrier.

**Fig. 5:**
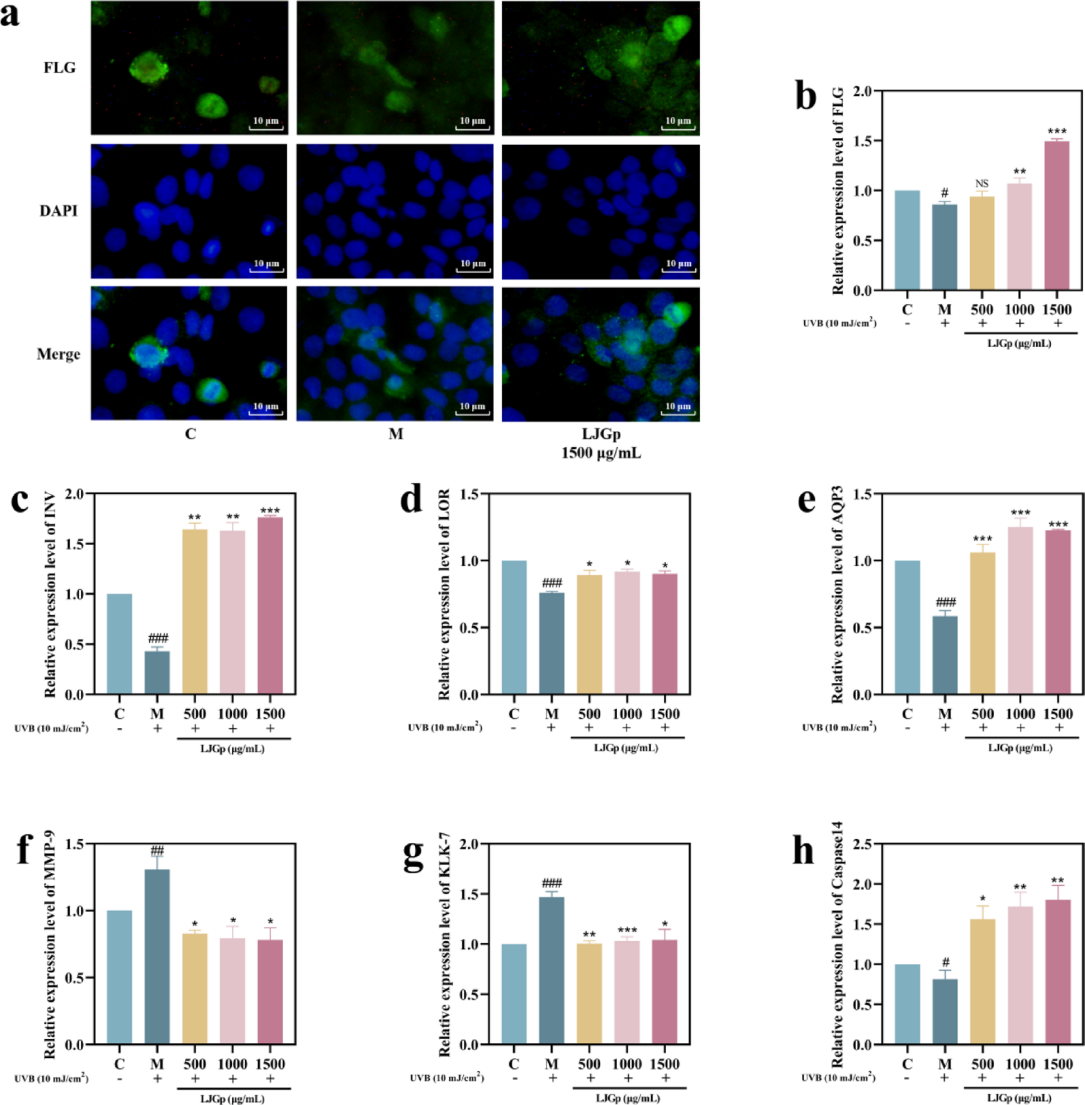
Effects of LJGp on the synthesis of barrier functional protein of HaCaT cells after UVB injury. **a** Immunofluorescence image of Filaggrin. Scale bars = 10 μm. **b** Filaggrin (FLG) content. **c** Involucrin (INV) content. (d) Loricrin (LOR) content. **e** Aquaporin 3 (AQP3) content. **f** Matrix metalloproteinase-9 (MMP-9) enzyme activity. **g** Kallikrein-7 (KLK-7) enzyme activity. **h** Caspase-14 enzyme activity. Statistical annotations: ***P < 0.001, **P < 0.01, *P < 0.05. Symbols ###, ##, # indicate comparisons with the model group

The effects of UVB on the activity of barrier function proteins and enzymes in epidermal cells, and the reparative effects of LJGp, are shown in Fig. 5. The IF results show that the undamaged FLG was oval with smooth edges. The fluorescence intensity of the model group histone decreased, and its shape was irregular and long. This morphological change suggests that FLG may lose its original physiological activity. In the sample group, the green bright oval protein fluorescence with clear edges could be seen around the nucleus. The ELISA results of FLG were similar to those of IF (Fig. 5a, b).

Otherwise, the contents of caspase-14, AQP3, FLG, INV, and LOR decreased to varying degrees, while the enzyme activities of KLK-7 and MMP-9 in the model group increased significantly. In the sample group, the enzyme activities of KLK-7 and MMP-9 decreased, while the enzyme activities of Caspase-14 increased. The contents of AQP3, FLG, INV, and LOR increased to varying degrees. Some indicators did not show a dose-dependent trend, but the effects of 1500 μg/mL concentration were the most significant overall (Fig. 5c-h).

These results confirm that UVB irradiation accelerated the disruption of intercellular bridging grain, increased the secretion of inflammatory mediators with proteolytic properties, and consumed a large amount of Caspase-14 and FLG to supplement the epidermal NMF. In addition to functional enzymes, UVB radiation also has a negative effect on the activity of epidermal barrier functional proteins. Fermented polysaccharides can increase the content of barrier functional proteins in the epidermal layer and inhibit the processes of desmosome shear, cell exfoliation, and water loss.

### LJGp regulates TRPV4-Keap-1/Nrf2 signaling pathway proteins and genes

The Keap-1/Nrf2 signaling pathway regulates including oxidation and inflammation. Its downstream factor AP-1 regulates the expression level of MMP-9, a barrier function protein hydrolase. The specific activation of TRPV4 by UVB and the potential regulation of ROS by the opening of calcium channels suggest that TRPV4 may form a complete triggering pathway with keap-1/Nrf2 signaling pathway.

**Fig. 6:**
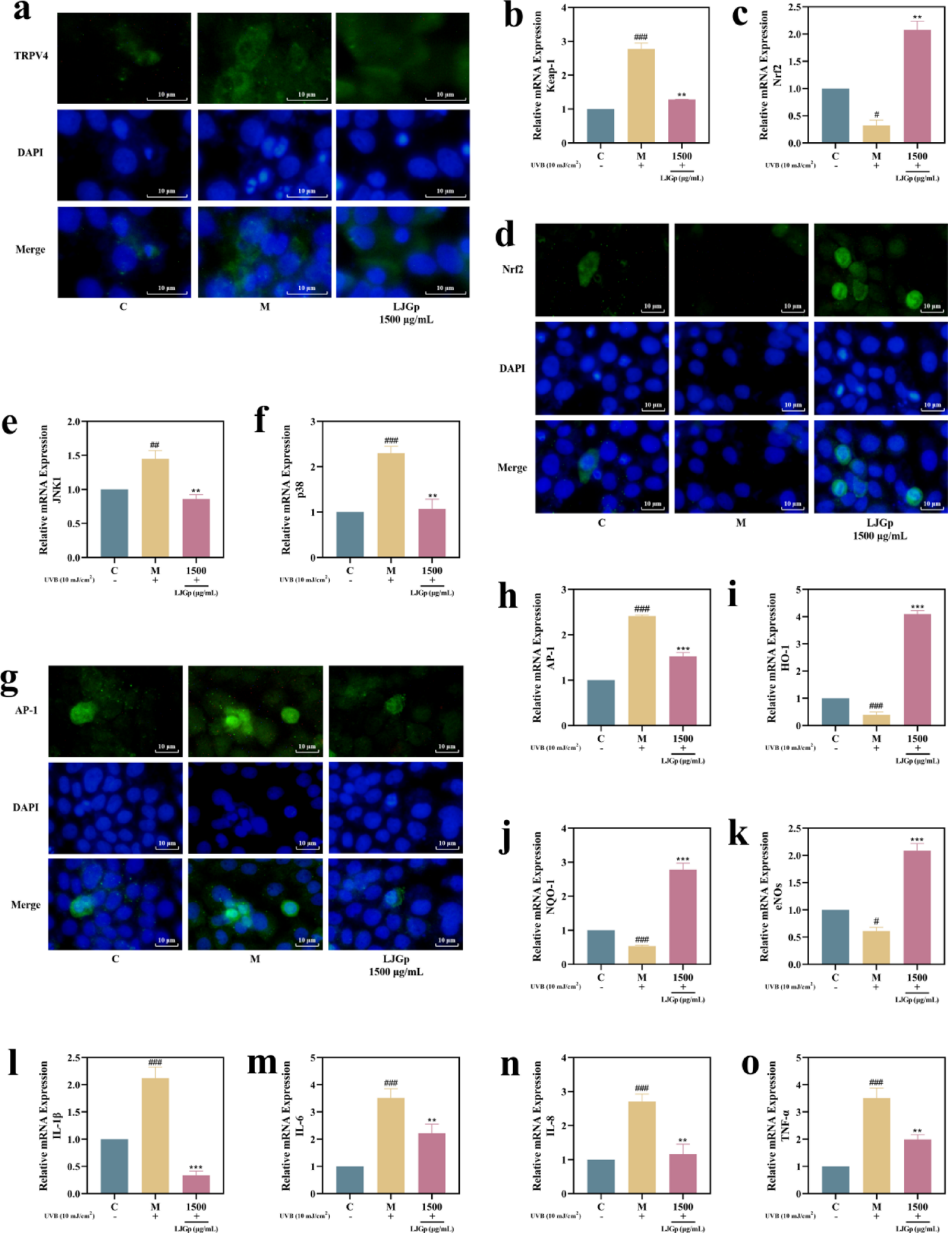
Effects of LJGp on TRPV4-Keap-1/Nrf2 signaling pathway related protein synthesis and gene transcription level. **a** Immunofluorescence image of TRPV4. Scale bars = 10 μm. **b** Keap-1 mRNA transcription levels. **c** Nrf2 mRNA transcription levels. **d** Nrf2 immunofluorescence image. Scale bars = 10 μm. **e** JNK1 mRNA transcription levels. **f** p38 mRNA transcription levels. **g** AP-1 immunofluorescence image. Scale bars = 10 μm. **h** AP-1mRNA transcription levels. **i** HO-1 mRNA transcription levels. **j** NQO-1 mRNA transcription levels. **k** eNOs mRNA transcription levels. **l** IL-1β mRNA transcription levels. **m** IL-6 mRNA transcription levels. **n** IL-8 mRNA transcription levels. **o** TNF-α mRNA transcription levels. Statistical annotations: ***P < 0.001, **P < 0.01, *P < 0.05. Symbols ###, ##, # indicate comparisons with the model group

Therefore, we detected the expression of key proteins and the transcription level of genes. The effects of UVB and LJGp on the TRPV4-Keap-1/Nrf2 signaling pathway-related protein synthesis transcription levels are shown in Fig. 6.

To determine whether fermented polysaccharides could exert regulatory effects on the initiating target, we evaluated the expression content of TRPV4 within HaCaT. Compared to the control group, the significant enhancement of TRPV4 fluorescence brightness was significantly increased in the model group. After sample treatment, the significant enhancement of the TRPV4 fluorescence brightness was significantly reduced compared with the model group, confirming that LJGp inhibited the high expression of TRPV4 after UVB irradiation (Fig. 6a).

The transcriptional levels of Keap-1 and Nrf2 are shown in Fig. 6b and c. When keratinocytes are damaged, Nrf2 needs to dissociate from Keap-1 and transfer into the nucleus to regulate the downstream genes to deal with the imbalance of homeostasis.

The IF images show that the green fluorescence of the Nrf2 protein was almost invisible in the model group. After treatment with LJGp, more green oval protein fluorescence was visible in the perinuclear region with smooth edges and clear morphology (Fig. 6d). The AP-1 protein fluorescence images showed opposite results to those of Nrf2. Compared with the control group, more green fluorescence with clear edges and elliptical morphology was seen around the nuclei in the model group. The green fluorescence was weakened after the action of LJGp, but still maintained its normal morphology (Fig. 6g). The IF results confirm that LJGp was able to alleviate the inactivation of Nrf2 induced by UVB irradiation and effectively inhibited the over-synthesis of AP-1 without affecting the active function of AP-1. Compared with the control group, the Keap-1, AP-1, JNK1, and p38 transcription levels in the model group were significantly increased, while the Nrf2 transcription levels were decreased. The transcription levels of each gene in the sample group showed the opposite trend. Except for Nrf2, the transcription levels of the other four genes with positive effects on oxidative damage in the sample group were the same as those in the control group (Fig. 6e, f, h–o).

The above results show that UVB irradiation stimulated the opening of the TRPV4 channel in epidermal cells and released Ca^2+^ into the cells. The separation process of the Keap-1-Nrf2 complex was inhibited, which activated the AP-1-mediated inflammatory response and promoted the hydrolysis process of barrier functional proteins. LJGp significantly inhibited the cellular inflammatory response induced by UVB irradiation through the TRPV4-Keap-1/Nrf2 cascade signaling pathway, and alleviated the loss of barrier function proteins. On the protein and molecular level, it effectively repaired light-induced epidermal photoinflammation and the weakening of the skin barrier function.

## Discussion

At present, research on skin inflammation mainly focuses on the development and efficacy verification of new drugs for inflammatory diseases such as AD and psoriasis, while research on the repair and blocking of epidermal cell damage in the early stages of inflammatory skin disease is still relatively weak (Biliński et al. 2025). In addition, the development focus of new targeted drugs has gradually shifted from synthetic chemicals to natural plant ingredients. *Angelica sinensis* extract has been shown to inhibit NF-κB-mediated centrocyte infiltration-induced AD via the Nrf2 signaling pathway, which provides new insight into allergic disease therapy (Lee et al. 2024). Icariin was able to specifically inhibit NLRP3 inflammatory vesicle-mediated pre-AD immune response by regulating the lncRNA MALAT1/miR-124-3p axis (Zhao et al. 2023). *Agaricus blazei* Murill polysaccharides reduce IL-1β and TNF-α while enhancing barrier proteins (Cheng et al. 2024), *Dendrobium nobile* polysaccharides protect HaCaT cells via antioxidant and MAPK-regulating pathways (Wang et al. 2025). Our previous studies have shown that Laminaria japonica Fermentation Broth was an inhibitor of epidermal photoinflammation with protective and restorative effects. Considering the combination of theoretical biological activity and practical use benefits, we extracted the polysaccharide components in LJGp (Sun et al. 2022).

The mechanistic results suggest that TRPV4 modulation and subsequent activation of the Keap1/Nrf2 pathway represent a key axis of LJGp action. While TRPV4 has been implicated in calcium signaling and inflammatory regulation in keratinocytes, evidence for its regulation by natural polysaccharides remains limited. Here, TRPV4 downregulation coincided with reduced ROS and enhanced Nrf2 translocation, pointing toward an indirect mechanism. Nevertheless, whether LJGp directly interacts with TRPV4 channels or secondarily modulates them through ROS reduction remains unresolved. Detailed functional assays, such as calcium influx measurements and TRPV4 antagonism studies, will be required to clarify this mechanism. It should be noted that the reduction of TRPV4 expression in this study was described qualitatively. Quantitative analyses such as Western blotting will be necessary to confirm the extent of TRPV4 modulation by LJGp and provide more precise mechanistic insights. Although multiple concentrations of LJGp (500–1500 μg/mL) were tested and showed dose-dependent protective effects, the physiological relevance of these concentrations in vivo requires further validation. Keratinocyte monolayer models, while useful for initial mechanistic insights, cannot fully reproduce the complexity of skin tissue (Wei et al. 2020). Since polysaccharide extracts often show batch-to-batch variation, their reproducibility and efficacy could be inconsistent, which makes standardization and quality control particularly important (Bai et al. 2023; Chadwick et al. 2025). Future in vivo animal experiments will also be needed to substantiate the protective effects of LJGp in a physiological context before considering clinical use. Future studies using 3D skin models or skin-on-a-chip systems are essential to confirm the skin-protective efficacy and safety of LJGp under physiological conditions (Lombardi et al. 2024).

From a translational perspective, LJGp shows promise as a cosmeceutical or dermatological ingredient, supported by its antioxidant, anti-inflammatory, and barrier-preserving effects. Future research should investigate formulation stability, skin penetration efficiency, and clinical efficacy, potentially in the context of sunscreen, after-sun repair, or sensitive-skin care formulations. In conclusion, this work identifies the TRPV4-Keap1/Nrf2 axis as a novel regulatory mechanism by which LJGp alleviates UVB-induced photodamage. This work not only provides mechanistic evidence for the developing inhibitors of inflammatory skin damage but also highlight the potential of polysaccharides obtained through this new extraction method, opening new avenues for their high-value utilization.

## Conclusion

The correlation between biological activity and substances was analyzed. LJGp, a polysaccharide component with strong correlation, was selected as the research object. UVB damage repair experiments have confirmed that it improves the survival rate of HaCaT and the activity of intracellular antioxidant enzymes, effectively inhibiting the production of oxygen free radicals and inflammatory factors. LJGp also inhibited the activity of intercellular junction cleavage enzymes and barrier structure protein-degrading enzymes, increasing the synthesis content of barrier functional proteins. In addition, LJGp also achieved the barrier function repair of radiation-damaged epidermis by regulating the key node genes at the beginning and middle of the TRPV4 Keap-1/Nrf2 signaling pathway. This study indicates that TRPV4-Keap-1/Nrf2 is a complete signaling pathway involved in the regulation of epidermal radiation damage. LJGp can exert photo-repair effects through the above pathways, alleviating epidermal inflammation and barrier dysfunction caused by UVB radiation.

## Supplementary Information

Below is the link to the electronic supplementary material.

Supplementary Material 1

## Data Availability

The data that support the findings of this study are available on request from the corresponding author Dongdong Wang, upon reasonable request.
