# Supplementary material for: Light repair effect of polysaccharides component from white Ganoderma lucidum and Laminaria japonica fermentation broth attenuates epidermal barrier dysfunction via TRPV4-Keap-1/Nrf2 pathway
Source: Bioresour Bioprocess. 2025 Oct 10;12(1):113. doi: 10.1186/s40643-025-00949-7 (PMC12514099; doi:10.1186/s40643-025-00949-7)
Supplement: Supplementary file 1 — Supplementary Material 1 [file 40643_2025_949_MOESM1_ESM.docx]

###### Table S1 Reverse transcription system.

| Reagent | Volume (μL) |
| --- | --- |
| Total RNA | 2.0 |
| Anchored Oligo(Dt)18 Primer | 1.0 |
| 2×ES Reaction Mix | 10.0 |
| EasyScript RT/RI Enzyme Mix | 1.0 |
| Gdna Remover | 1.0 |
| Rnase-free Water | 5.0 |

###### Table S2 Primer sequences for Real-Time PCR

| Gene | Direction | Primer sequence (5 '→ 3') |
| --- | --- | --- |
| TNF-α | F  R | CACAGTGAAGTGCTGGCAAC  AGGAAGGCCTAAGGTCCACT |
| Keap-1 | F  R | GGAGGCGGAGCCCGA  GATGCCCTCAATGGACACCA |
| HO-1 | F | CAAGCGCTATGTTCAGCGAC |
|  | R | GCTTGAACTTGGTGGCACTG |
| IL-1β | F | CCTGAGCTCGCCAGTGAAA |
|  | R | GTGGTGGTCGGAGATTCGTA |
| AP-1 | F  R | TCTCAACATGGGTGGTCTGT  AAATGCTTCATGCGGCGAAG |
| IL-8 | F  R | AAGATGTGAAGCTGACGCAGA  AGAATTGAGCTGAGCCTTGG |
| MMP-9 | F  R | GTACTCGACCTGTACCAGCG  AGAAGCCCCACTTCTTGTCG |
| JNK1 | F  R | CTGTGTGGAATCAAGCACCTTCA  CTGGCCAGACCGAAGTCAAGA |
| p38 | F  R | TTAACAGGATGCCAAGCCATGA  GGCACCAATAAATACATTCGCAAAG |
| Nrf2 | F  R | CAACTCAGCACCTTGTATC  TTCTTAGTATCTGGCTTCTT |
| NQO1 | F  R | CAGCCAATCAGCGTTCGGTA  CTTCATGGCGTAGTTGAATGATGTC |
| eNOS | F | GCCGGAACAGCACAAGAGTT |
|  | R | CTCCGTTTGGGGCTGAAGAT |
| β-action | F | TGGCACCCAGCACAATGAA |
|  | R | CTAAGTCATAGTCCGCCTAGAAGCA |

F: forward primer; R: reverse primer.

###### Table S3 Reagents and dosage.

| Reagent | Volume (μL) |
| --- | --- |
| Template | 1.5 |
| Forward Primer (10 μM) | 0.4 |
| Reverse Primer (10 μM) | 0.4 |
| 2×TransStart® Top Green qPCR SuperMix | 10.0 |
| Passive Reference Dye (50×) | 0.4 |
| Nuclease-free Water | 7.3 |
